# Distinct Effects of Stimulus Repetition on Various Temporal Stages of Subject’s Own Name Processing

**DOI:** 10.3390/brainsci12030411

**Published:** 2022-03-21

**Authors:** Yihui Zhang, Musi Xie, Yuzhi Wang, Pengmin Qin

**Affiliations:** 1 Key Laboratory of Brain, Cognition and Education Sciences, Ministry of Education, South China Normal University, Guangzhou 510631, China; zhangyihui@m.scnu.edu.cn (Y.Z.); xiemusi@163.com (M.X.); 2 Center for Studies of Psychological Application, School of Psychology, South China Normal University, Guangzhou 510631, China; 3 Guangdong Key Laboratory of Mental Health and Cognitive Science, South China Normal University, Guangzhou 510631, China; 4 Department of Western Medicine Surgery, Shandong University of Traditional Chinese Medicine, Jinan 250355, China; wangyuzhi_qin@163.com; 5 Pazhou Lab, Guangzhou 510335, China

**Keywords:** subject’s own name, event-related potential (ERP), LPP, self-processing

## Abstract

The self is one of the most important concepts in psychology, which is of great significance for human survival and development. As an important self-related stimulus, the subject’s own name (SON) shows great advantages in cognitive and social processing and is widely used as an oddball stimulus in previous studies. However, it remained unknown whether the multiple repetition of stimulus would have similar influence on the neural response to SON and the other names under equal probability. In this study, adopting EEG and an equal–probability paradigm, we first detected the SON-related ERP components which could differentiate SON from other names, and then investigated how these components are influenced by repeated exposure of the stimulus. Our results showed that SON evoked an earlier SON-related negativity (SRN) at the fronto-central region and a late positive potential (LPP) at the centro-parietal region. More intriguingly, the earlier SRN demonstrated reduction after multiple repetitions, whereas LPP did not exhibit significant changes. In conclusion, these findings revealed that multiple repetitions of the stimulus might influence the various temporal stages in SON-related processing and highlighted the robustness of the late stage in this processing.

## 1. Introduction

The self is one of the most important concepts in psychology, which is of great significance for human survival and development. Previous literature has revealed cognitive advantages (faster and more accurate responses) in the processing of self-related information (such as one’s own name or own face) [1,2,3], which could be used for higher-level sensory integration and eventually creating an intact representation of the self [4,5]. Subject’s own name (SON) is commonly used to evoke a self-related neural response in a wide range of research works [6,7,8,9,10]. More importantly, the self-related neural response evoked by hearing one’s own name could be distinct in the temporal domain. For example, previous event-related potential (ERP) studies found that hearing the subject’s own name could evoke an SON negativity in 170–270 ms over the fronto-central area [11,12,13], a larger P3 amplitude at around 300 ms [11,14,15,16,17,18,19,20,21] relative to other names, and a late positive potential (LPP) at around 400–800 ms, which was proposed to be associated with the recollection of self-related and other-related information [22]. The above findings suggested that the distinct neural responses to SON may occur in different stages of SON processing. However, most previous research employed SON as an oddball stimulus to evaluate their brain response [9,11,12,14,15], in which the novelty could present confounding factors to the neural properties of SON. Therefore, it is still uncertain which components could distinguish the SON from other names under equal probability.

The neural response evoked by a stimulus was proposed to be influenced by repetition. Previous research found that repetitions of stimuli could affect neural responses to the input, either in visual or in auditory tasks [23,24,25]. Moreover, such effects could be reflected in ERP activation patterns, including early negative [26] and late positive potentials [24], which makes it an appropriate approach in investigating the differential responses of SON processing.

To achieve this aim, the current study carried out an EEG experiment on healthy participants, using three names as auditory stimuli presented with the same probability: SON, a friends’ name (FN), and an unknown name (UN). Specifically, we first compared the ERP components of SON to those of other names to identify the SON-related components. Then, to investigate the impact of repetitions on SON, we divided all trials in half and compared SON-related components between the first and second halves of the trials.

## 2. Materials and Methods

### 2.1. Participants

Twenty-six healthy adult participants were recruited and compensated for their participation in this experiment (13 males, and 13 females, ages between 18 and 26 years, Mean = 20.54, SD = 2.08). All subjects were right-handed, had normal hearing, and spoke Chinese as their native language. All subjects reported no history of mental disorders. Informed consent was obtained from each subject before the study. This study was approved by the ethics committee of the School of Psychology in South China Normal University.

### 2.2. Stimuli

Four types of auditory stimuli were used: subject’s own name (SON), a friend’s name (FN), an unknown name (UN), and an English name (EN). For FN, to minimize the confounding effect of both a high self-relatedness and familiarity in a close other’s name, such as parent’s name, as used in many previous studies [6,11,27], we chose to use classmates’ name instead, which has a moderate familiarity and low self-relatedness. To control the physical features of SON and UN at the group level, the UN used for each subject was randomly selected from other subjects’ names. Only names of the same gender as the subject were used for FN and UN. All voice recordings were edited at a sampling rate of 44 100 Hz and 16-bit resolution, which were standardized by peak amplitudes [6]. All names were disyllables. The names were read by a native Chinese speaker, who was an adult female unknown to the subjects. The names were presented binaurally using headphones at 75 dB with E-prime 3.0 (Psychology Software Tools, Inc., Sharpsburg, Pennsylvania, USA). The duration of the names was between 490 and 777 ms (Mean = 577.62, SD = 51.72), with the second syllable starting at around 230 ms, on average.

### 2.3. Experimental Procedure

**Figure 1:**
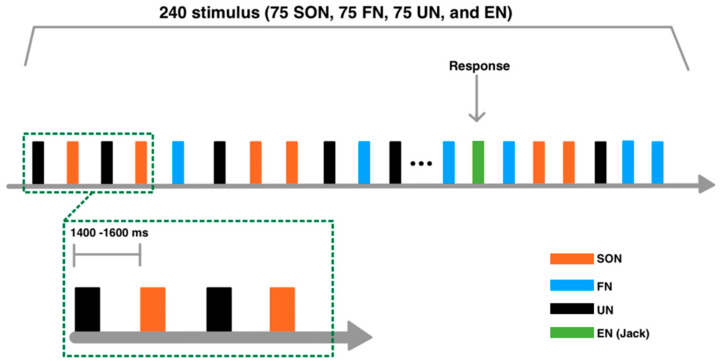
Experimental paradigm. A total of 960 trials were presented in four blocks, each consisting of 240 trials. Subjects were required to keep their eyes open and fixate on a white cross displayed on a black screen. In all blocks, subjects listened to the names and pressed the button with their right index finger when they heard JACK. Four types of auditory stimuli were used: subject’s own name (SON), a friend’s name (FN), an unknown name (UN), and an English name (EN: JACK).

All stimuli were randomly presented. In order to maintain the subjects’ attention, 15 repetitions of the English name (EN: JACK) were interspersed randomly. The stimulus onset asynchrony (SOA) duration varied randomly from 1400 to 1600 ms. A total of 960 trials were presented in four blocks, each consisting of 240 trials (75 SON, 75 FN, 75 UN, and 15 EN), separated by a short break (Figure 1). Subjects were required to keep their eyes open and fixate on a white cross displayed on a black background screen. In all blocks, subjects listened to the names and pressed the button with their right index finger when they heard JACK.

### 2.4. EEG Recordings

The EEG signals were continuously recorded using a BrainVision Recording (Brain Products GmbH, Munich, Germany) from 32 scalp electrodes that were mounted on an elastic cap according to the 10–20 system. All channels were referenced to the FCz online. The electrode impedance was kept below 5 kΩ. To monitor eye movements, a vertical electrooculogram was also recorded from the electrodes placed below the left eye. The EEG signals were recorded with a sampling rate of 1000 Hz and band-pass filtered online at 0.01–100 Hz. Each recording session lasted 60 min, including cap electrode preparation. To relieve the subjects from weariness, short breaks were provided after each set of trials. Each block lasted approximately 6 min.

### 2.5. Electroencephalogram Analysis

EEG data analysis was performed in MATLAB (The MathWorks, MA) using the EEGlab (http://www.sccn.ucsd.edu/eeglab (accessed on 5 January 2022)) and Letswave toolbox (https://letswave.cn/ (accessed on 5 January 2022)). The continuous data were filtered offline using a band-pass filter from 0.1 to 30 Hz (6dB/octave) with a basic FIR filter. The filtered data were then re-referenced to contralateral mastoids. A total of 100 ms of pre-stimulus epochs and 900 ms of post-stimulus epochs were extracted. Baseline correction was then applied to the pre-stimulus interval. Trials contaminated by eye movements, eye blinks, or muscle potentials were rejected before averaging. On average, 11% of the trials were rejected. The mean acceptance rates of the remaining epochs were 89.26% for SON, 88.69% for FN, and 90.30% for UN. One-way ANOVA showed no significant difference in the acceptance rates (F (2, 69) = 0.226, *p* = 0.798).

For the ERP data analysis, we first identified the time windows (TW) for significant SON effects by an exploratory approach (i.e., sliding time window). Specifically, we calculated the grand average of ERP waveforms across trials and participants in each name condition (SON, FN and UN). Then two pairwise comparisons (SON-FN and SON-UN) were carried out with a sliding time window of 20 ms and steps of 1 ms at each electrode [28]. Two *p*-value matrices of electrodes × time points were obtained. Assessment of statistical significance for these two matrices were based on cluster-based permutation *t*-tests. The type I error rate was controlled by evaluating the cluster-level statistics under a randomized null distribution of the maximum cluster-level statistics, which could be determined by randomly shuffling the condition labels 1000 times. In this way, the time windows with significant SON effects were found.

Within each TW, the scalp topography of each name condition (SON, FN, UN) and pairwise comparison (SON-FN, SON-UN) were plotted, and the three electrodes with the greatest difference in the two pairs of comparisons were selected for further analysis. The grand average of ERP waveforms across these electrodes were then calculated for each name condition, and the mean amplitude of these waveforms within each TW were compared between conditions using repeated-measures ANOVA and post hoc comparisons. Additionally, in order to further identify the SON-related effects, we subtracted the waveforms of other names (UN and FN) from SON to obtain the difference waves (i.e., SON-FN and SON-UN), which reflected a self-specific effect.

Furthermore, to explore how SON-related effects were influenced by the repeated exposure of the stimuli, we tested the variations of multiple repetitions. To do so, we first split all trials into two halves. In each half of the trials, we averaged the amplitude of difference waves (SON-UN, SON-FN) within each TW and across trials, generating the mean amplitude of SON-related effects for each participant. Then the mean amplitude of SON-related effects was compared between the two halves of trials using the paired *t*-test.

To rule out the confounding effect of familiarity in the mean amplitude difference between the first and second halves of trials, we analyzed the variations of difference between two halves of the trials for the SON, FN, and UN conditions, respectively. Two-way repeated measures ANOVA with names (SON, FN, UN) and trials (first and second half) as factors and post hoc paired comparison were conducted within each TW of the SON-related effects. In addition, to identify the topographic distribution of the SON-related effects, additional statistical analyses were performed (see Supplementary Material for details).

## 3. Results

**Figure 2:**
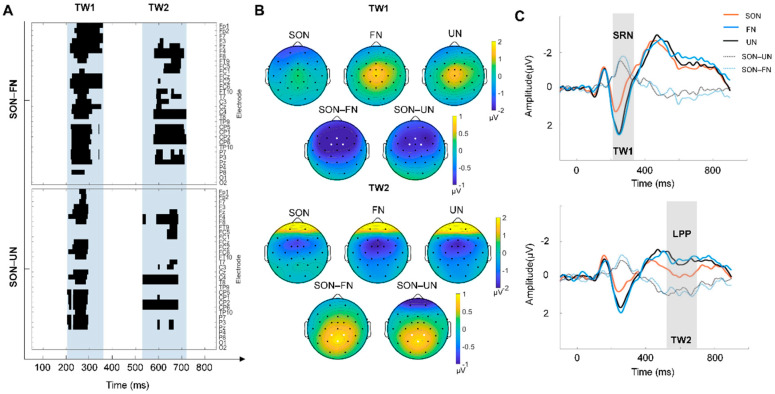
The result of the SON-related effect. (**A**) P-value matrices of electrodes × time points obtained from two pairwise comparisons (Top: SON–FN, Bottom: SON–UN), which were carried out with a sliding time window of 20 ms and steps of 1 ms at each electrode. The blue shaded area indicated two time windows (TW) with significant differences (cluster corrected): TW1 (210–360 ms) and TW2 (520–720 ms). (**B**) The topographic map illustrates the scalp distribution of ERP amplitudes of each condition and the difference between SON and other names (FN/UN) in TW1 (top), and TW2 (bottom). The electrodes with the greatest difference are marked with white dots: Fz, FC1, FC2 for TW1, Pz, CP1 and CP2 for TW2. (**C**) The grand averages of ERPs across Fz, FC1, and FC2 electrodes within TW1 are shown on the top. SRN stands for self-related negativity, which is the SON-evoked negative component. Grand averages of ERPs across channels Pz, CP1, and CP2 electrodes within TW2 are shown at the bottom. LPP stands for late positive potential, which is the SON-evoked positive component.

According to the sliding time window, two TWs with a significant SON effect were found: (1) TW1: 210–360 ms and (2) TW2: 520–720ms (Figure 2A). The corresponding scalp topography of TWs were plotted, and three electrodes with the greatest difference were marked with white dots in each TW topography: Fz, FC1, FC2 for TW1, and Pz, CP1 and CP2 for TW2 (Figure 2B). The repeated-measures ANOVA of the mean amplitude within TWs showed a significant main effect (TW1: F (2, 50) = 13.795, *p* < 0.001. TW2: F (2, 50) = 13.488, *p* < 0.001). Post hoc comparison further revealed that, compared to that of the FN and UN condition, the mean amplitude in the SON condition was more negative within TW1 (*p* < 0.01 Bonferroni correction) and more positive within TW2 (*p* < 0.01, Bonferroni correction) (Figure 2C). The result of the difference wave analysis identified a SON-related negativity (SRN) in TW1 (210–360 ms) and late positive potential (LPP) in TW2 (520–720 ms) (Figure 2C). In addition, no difference between FN and UN in the post hoc comparison was found.

**Figure 3:**
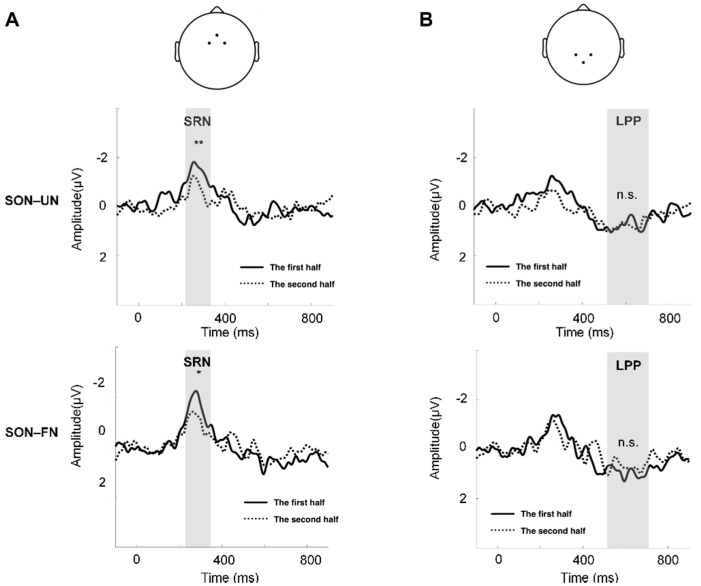
The comparison of SON-related effect between the first and second half of the trials. (**A**) The channels to compute the mean waveforms of SRN: Fz, FC1, and FC2 (top). The comparison of SON–UN waveforms between the two halves of trials (middle). The comparison of SON–FN waveforms between the two halves of trials (bottom). The gray shading indicates TW1. (**B**) The channels to compute the mean waveforms of LPP: Pz, CP1, and CP2 (top). The comparison of SON–UN waveforms between the two halves of trials (middle). The comparison of SON–FN waveforms between the two halves of trials (bottom). The gray shading indicates TW2. ** = *p* < 0.01, * = *p* < 0.05, n.s. = *p* > 0.05.

The paired *t*-test between the two halves of trials showed decreased amplitudes of SRN in the second half trials, compared to the first half trials (SON-UN: t = 3.445, *p* < 0.01; SON-FN: t = 2.334, *p* < 0.05) (Figure 3A). No significant difference of amplitude was found in LPP between the two halves of trials (Figure 3B).

**Figure 4:**
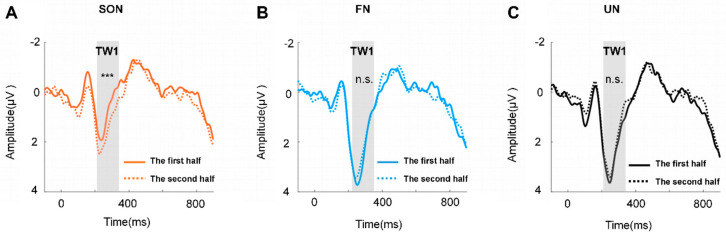
The grand average waveform across the channel of TW1 (Fz, FC1, FC2). (**A**) The grand average waveform of SON between the two halves of trials. (**B**) The grand average waveform of FN between the two halves of trials. (**C**) The grand average waveform of UN between the two halves of trials. *** = *p* < 0.001, n.s. = *p* > 0.05.

Furthermore, the two-way repeated measures ANOVA of TW1 exhibited significant main effects of names (F (2, 50) = 5.774, *p* < 0.01, η^2^p = 0.188) and trials (F (1, 25) = 10.111, *p* < 0.01, η^2^p = 0.288), and a significant interaction effect between these two factors (F (2, 50) = 7.724, *p* < 0.01, η^2^p = 0.236), but no significant effect was found in TW2. The post hoc paired comparison of TW1 revealed a significant difference only in the SON condition (F (2, 50) = 19.413, *p* < 0.001, η^2^p = 0.437), but not in FN or UN (Figure 4).

## 4. Discussion

To summarize, the current study investigated the SON-related ERP components and the influence of stimulus repetition on these components in an equal probability paradigm. In our results, rather than in other names (FN and UN), the SRN (210–360 ms) at fronto-central regions and LPP (520–720 ms) at centro-parietal regions were only found in the SON condition. Moreover, the SRN showed a significant reduction in the second half of trials as compared to the first half. Taken together, we found that stimulus-repetition could affect different stages in the processing of SON.

The most interesting finding in the current study is that the LPP evoked by SON in the centro-parietal region was not affected by repetition. This is supported by the finding that LPP is a robust ERP component over a variety of paradigms, both visual and auditory [6,14,22]. Furthermore, the LPP in the centro-parietal region has been proposed to be associated with processing information of personal significance [6], episodic memory recollection, personal identity and semantic information [29]. Since the physical properties of the stimuli (SON and UN) were rigorously controlled in our experiment, the preservation of LPP under repetitions of the SON could reflect a stable representation of the self in the late stages of mental processing.

In addition, the SRN in the fronto-central region was reduced by the repetitions of SON, which might be related to the repetition suppression effect. This finding was supported by an fMRI study showing a repetition suppression effect on the ventromedial prefrontal cortex (vMPFC) during self-related processing [30]. According to the classic conflict theory, the component in the fronto-central region within 200–300 ms mainly reflects stimulus-related conflict in the oddball paradigm, and the most widely proposed interpretation of this component is related to mismatch at the stimulus level and attentional shifts [14,19,31,32]. However, the situation is different when the stimulus is presented with equal probability. When cognitive conflict is absent or when there is no novel stimulus, this component in the fronto-central area reflects more stimulus-related cognitive processing [33,34,35]. Furthermore, predictive coding theory may also provide a possible interpretation for this suppression effect [4,5,36,37]. According to this theory, the bottom–up auditory signals could be iteratively compared with the top–down predictions of self-relatedness during the repetition, which resulted in a dynamic change in the internal prediction of self-relatedness to minimize prediction errors. This implies that the decreased SRN amplitudes may reflect the reduction in prediction errors, which might enhance the suppression of repeated stimuli. It is worth noticing that the variations of repetition were only yielded by SON rather than other names within the TW of SRN, suggesting that SRN is sensitive to self-relatedness rather than familiarity. With both the onset time (around the start of second syllables) and the suppression effect considered, the SRN found in the current study may reflect the representation of self-specific information.

Notably, the SRN found in the current study is different from the SON negativity (170–270 ms) which was evoked by the SON found in the oddball paradigm [11]. The difference between them is mainly reflected in two aspects. First, the SRN (210–360 ms) occurred later than SON negativity. Second, it showed reduction under stimulus repetition, whereas SON negativity did not. These two aspects might be due to the experiment design. Specifically, the SRN evoked in the current study was found using an equal-probability paradigm and an active task, while the SON negativity found in previous studies occurred in an oddball paradigm and passive tasks, which require subjects to play games and ignore the auditory stimulus [11,12]. Taken together, the evidence implied that the repetition of external self-related stimulus could affect self-related processing in a complicated way, and further investigation is needed to rule out confounding factors, such as the number of repetitions.

Lastly, the dissociation between SON and FN calls for further attention. Previous EEG studies obtained inconsistent results regarding the relationship between self-processing and familiarity. Some research studies found that self-processing shares some similarities with familiarity processing [11,17,27,38], while others found otherwise [39]. There are two possible reasons for these inconsistencies. First is the choice of FN (e.g., parents’ or close friends’ names often have both high familiarity and self-relatedness). In the current study, FN was selected from classmates with a moderate level of familiarity and low self-relatedness, which may explain why FN causes similar ERP response as the UN rather than SON, which could provide further evidence that the SRN component is specific to self-relatedness rather than to familiarity. On the other hand, these inconsistencies could also come from differences in experimental paradigms. In previous oddball paradigm studies on familiarity, names were used as low frequency stimulus [6,40], which might cause different levels of saliency, inducing attentional shifts [11]. In the current study however, we used an equal probability paradigm instead, which could result in a higher similarity of responses between familiar names and other names. Further studies are required to clarify these inconsistencies.

## 5. Conclusions

In the current study, we employed the electrophysiological method with a high temporal resolution to investigate how SON-related ERP components are influenced by repeated exposure of the stimuli. Our results showed that SON could evoke two SON-related ERP components: the earlier SRN at the fronto-central region and the later LPP at the centro-parietal region. More interestingly, the results suggested that stimulus repetition could affect various stages of self-related processing, in which repetition suppression could emerge in the earlier stage of SON-related processing (SRN), but not the later stage (LPP). These results primarily highlight the robustness of the late stage of SON-processing.

## Data Availability

The data that support the findings of this study are available on request from the corresponding author. The data are not publicly available due to privacy restrictions.

## Supplementary Materials

Some additional statistical analyses were done to identify the topographic distribution of the SON-related effects. The following supporting information can be downloaded at: https://www.mdpi.com/article/10.3390/brainsci12030411/s1.

## References

[1] Bortolon C., Raffard S. (2018). Self-face advantage over familiar and unfamiliar faces: A three-level meta-analytic approach. Psychon. Bull. Rev..

[2] Yang H., Wang F., Gu N., Gao X., Zhao G. (2013). The cognitive advantage for one’s own name is not simply familiarity: An eye-tracking study. Psychon. Bull. Rev..

[3] Keyes H., Brady N., Reilly R.B., Foxe J.J. (2010). My face or yours? Event-related potential correlates of self-face processing. Brain Cogn..

[4] Apps M.A., Tsakiris M. (2014). The free-energy self: A predictive coding account of self-recognition. Neurosci. Biobehav. Rev..

[5] Seth A.K. (2013). Interoceptive inference, emotion, and the embodied self. Trends Cogn. Sci..

[6] Eichenlaub J.-B., Ruby P., Morlet D. (2012). What is the specificity of the response to the own first-name when presented as a novel in a passive oddball paradigm? An ERP study. Brain Res..

[7] Cavinato M., Volpato C., Silvoni S., Sacchetto M., Merico A., Piccione F. (2011). Event-related brain potential modulation in patients with severe brain damage. Clin. Neurophysiol..

[8] Kempny A.M., James L., Yelden K., Duport S., Farmer S., Playford E.D., Leff A. (2018). Patients with a severe prolonged Disorder of Consciousness can show classical EEG responses to their own name compared with others’ names. NeuroImage Clin..

[9] Qin P., Di H., Yan X., Yu S., Yu D., Laureys S., Weng X. (2008). Mismatch negativity to the patient’s own name in chronic disorders of consciousness. Neurosci. Lett..

[10] Blume C., del Giudice R., Lechinger J., Wislowska M., Heib D.P., Hoedlmoser K., Schabus M. (2017). Preferential processing of emotionally and self-relevant stimuli persists in unconscious N2 sleep. Brain Lang..

[11] Tateuchi T., Itoh K., Nakada T. (2012). Neural mechanisms underlying the orienting response to subject’s own name: An event-related potential study. Psychophysiology.

[12] Tateuchi T., Itoh K., Nakada T. (2015). Further characterization of “subject’s own name (SON) negativity”, an ERP component reflecting early preattentive detection of SON. BMC Res. Notes.

[13] Thomas R.P., Wang L.A.L., Guthrie W., Cola M., Mccleery J.P., Pandey J., Schultz R.T., Miller J.S. (2019). What’s in a name? A preliminary event-related potential study of response to name in preschool children with and without autism spectrum disorder. PLoS ONE.

[14] Holeckova I., Fischer C., Giard M.-H., Delpuech C., Morlet D. (2006). Brain responses to a subject’s own name uttered by a familiar voice. Brain Res..

[15] Holeckova I., Fischer C., Morlet D., Delpuech C., Costes N., Mauguière F. (2008). Subject’s own name as a novel in a MMN design: A combined ERP and PET study. Brain Res..

[16] Li R., Song W., Du J., Huo S., Shan G. (2015). Electrophysiological correlates of processing subject’s own name. NeuroReport.

[17] Fan W., Chen J., Wang X.-Y., Cai R., Tan Q., Chen Y., Yang Q., Zhang S., Wu Y., Yang Z. (2013). Electrophysiological Correlation of the Degree of Self-Reference Effect. PLoS ONE.

[18] Fan X., Han S. (2018). Neural responses to one’s own name under mortality threat. Neuropsychologia.

[19] Wang X.-Y., Wu H.-Y., Lu H.-T., Huang T.-T., Zhang H., Zhang T. (2017). Assessment of mismatch negativity and P300 response in patients with disorders of consciousness. Eur. Rev. Med. Pharmacol. Sci..

[20] Niu G., Yao L., Kong F., Luo Y., Duan C., Sun X., Zhou Z. (2020). Behavioural and ERP evidence of the self-advantage of online self-relevant information. Sci. Rep..

[21] Doradzińska Łucja, Wójcik M.J., Paź M., Nowicka M.M., Nowicka A., Bola M. (2020). Unconscious perception of one’s own name modulates amplitude of the P3B ERP component. Neuropsychologia.

[22] Tamura K., Mizuba T., Iramina K. (2016). Hearing subject’s own name induces the late positive component of event-related potential and beta power suppression. Brain Res..

[23] Ferrari V., Bradley M.M., Codispoti M., Lang P.J. (2015). Massed and distributed repetition of natural scenes: Brain potentials and oscillatory activity. Psychophysiology.

[24] Ferrari V., Mastria S., Codispoti M. (2020). The interplay between attention and long-term memory in affective habituation. Psychophysiology.

[25] Jäncke L., Kühnis J., Rogenmoser L., Elmer S. (2015). Time course of EEG oscillations during repeated listening of a well-known aria. Front. Hum. Neurosci..

[26] Engell A., McCarthy G. (2014). Repetition suppression of face-selective evoked and induced EEG recorded from human cortex. Hum. Brain Mapp..

[27] Nijhof A.D., Dhar M., Goris J., Brass M., Wiersema J.R. (2018). Atypical neural responding to hearing one’s own name in adults with ASD. J. Abnorm. Psychol..

[28] Ding Y., Martinez A., Qu Z., Hillyard S.A. (2013). Earliest stages of visual cortical processing are not modified by attentional load. Hum. Brain Mapp..

[29] Tacikowski P., Jednoróg K., Marchewka A., Nowicka A. (2011). How multiple repetitions influence the processing of self-, famous and unknown names and faces: An ERP study. Int. J. Psychophysiol..

[30] Jenkins A.C., Macrae C.N., Mitchell J.P. (2008). Repetition suppression of ventromedial prefrontal activity during judgments of self and others. Proc. Natl. Acad. Sci. USA.

[31] Tomé D., Barbosa F., Nowak K., Marques-Teixeira J. (2014). The development of the N1 and N2 components in auditory oddball paradigms: A systematic review with narrative analysis and suggested normative values. J. Neural Transm..

[32] Folstein J.R., Van Petten C. (2007). Influence of cognitive control and mismatch on the N2 component of the ERP: A review. Psychophysiology.

[33] Berchicci M., Spinelli D., Di Russo F. (2016). New insights into old waves. Matching stimulus- and response-locked ERPs on the same time-window. Biol. Psychol..

[34] Sokhadze E.M., Casanova M.F., Casanova E., Lamina E., Kelly D.P., Khachidze I. (2017). Event-related Potentials (ERP) in Cognitive Neuroscience Research and Applications. NeuroRegulation.

[35] Conley E. (1999). The N100 auditory cortical evoked potential indexes scanning of auditory short-term memory. Clin. Neurophysiol..

[36] Qin P., Wang M., Northoff G. (2020). Linking bodily, environmental and mental states in the self—A three-level model based on a meta-analysis. Neurosci. Biobehav. Rev..

[37] Summerfield C., Trittschuh E.H., Monti J.M., Mesulam M.-M., Egner T. (2008). Neural repetition suppression reflects fulfilled perceptual expectations. Nat. Neurosci..

[38] Key A.P., Jones D., Peters S.U. (2016). Response to own name in children: ERP study of auditory social information processing. Biol. Psychol..

[39] Hirata S., Matsuda G., Ueno A., Fuwa K., Sugama K., Kusunoki K., Fukushima H., Hiraki K., Tomonaga M., Hasegawa T. (2011). Event-Related Potentials in Response to Subjects’ Own Names: A Comparison between Humans and a Chimpanzee. Commun. Integr. Biol..

[40] Zhu S., Long Q., Li X., Yang J., Li H., Yuan J., Long Q. (2018). Self-relevant processing of stranger’s name in Chinese society: Surname matters. Neurosci. Lett..

