# Supplementary material for: Distinct Effects of Stimulus Repetition on Various Temporal Stages of Subject’s Own Name Processing
Source: Brain Sci. 2022 Mar 21;12(3):411. doi: 10.3390/brainsci12030411 (PMC8946540; doi:10.3390/brainsci12030411)
Supplement: Supplementary file 1 [file brainsci-12-00411-s001.zip › brainsci-1606231-supplementary.pdf]

Supplementary Information

Distinct effects of stimulus repetition on various temporal stages of subject's own name processing

Yihui Zhang[a#], Musi Xie[a#], Yuzhi Wang[b], Pengmin Qin[a,c]*

Additional statistical details

To provide more detailed information, we also performed additional statistical analysis.

SRN

The mean amplitudes within the SON-related negativity time windows for each condition went through a three-way repeated measures ANOVA with names (SON, FN, UN), trials (first and second half), and cortical areas (fronto-central and centro-parietal EEG electrodes) as factors. The results showed significant main effects of names (F (2, 50) = 11.352, $p < 0.001$, η 2p = 0.312) and cortical areas (F (1, 25) = 5.828, $p < 0.05$, η 2p = 0.189). The mean amplitude in the SON condition was more negative than in FN ($p < 0.001$) and UN ($p < 0.01$) after Bonferroni correction. The mean amplitude of fronto-central EEG electrodes was larger than that of centro-parietal EEG electrodes, indicating that the SRN was stronger at the fronto-central EEG electrodes. A significant interaction effect was found between names and trials (F (2, 50) = 5.550, $p < 0.01$, η 2p = 0.182). Post-hoc comparisons (Bonferroni correction) further revealed that, for SON, the mean amplitudes in the first half were more negative than in the second half ($p < 0.05$).

LPP

The mean amplitudes within the LPP time windows for each condition were submitted to a two-way repeated measures ANOVA with names (SON, FN, UN) and cortical areas (fronto-central and centro-parietal EEG electrodes) as factors. The results showed significant main effects of names (F (2, 50) = 9.169, $p < 0.001$, η 2p = 0.268) and

cortical areas ($F(1, 25) = 20.359$, $p < 0.001$, $\eta 2p = 0.449$). The mean amplitudes of centro-parietal EEG electrodes were more positive than that of fronto-central EEG electrodes in SON ($p < 0.001$), FN ($p < 0.001$) and UN ($p < 0.01$) conditions after Bonferroni correction.
